# A Comparison of the Condyle and Articular Eminence in Asian Juvenile Idiopathic Osteoarthritis Patients with Unilateral and Bilateral TMJ Involvement: A Retrospective Case-Control Study

**DOI:** 10.3390/jcm12175566

**Published:** 2023-08-26

**Authors:** Hye-Min Ju, Hee-Won Kim, Seo-Young Choi, Hye-Mi Jeon, Sung-Hee Jeong, Yong-Woo Ahn, Soo-Min Ok

**Affiliations:** 1 Department of Oral Medicine, School of Dentistry, Pusan National University, Dental and Life Science Institute, Yangsan-si 50612, Republic of Korea; jc2wma@pusan.ac.kr (H.-M.J.); drcookie@pusan.ac.kr (S.-H.J.); ahnyongw@pusan.ac.kr (Y.-W.A.); 2 Department of Oral Medicine, Pusan National University Dental Hospital, Dental Research Institute, Yangsan-si 50612, Republic of Korea; yuys0927@naver.com (H.-W.K.); csycsy2004@naver.com (S.-Y.C.); 3 Dental Clinic Center, Pusan National University Hospital, Busan 49241, Republic of Korea; hyemicon@hanmail.net

**Keywords:** juvenile idiopathic osteoarthritis, temporomandibular joint, conebeam CT, condyle, articular eminence

## Abstract

This study compared the condylar volume, length, and articular eminence (AE) characteristics of normal individuals to those with unilateral and bilateral juvenile idiopathic osteoarthritis (JOA). The 116 patients were divided into four groups: Control (n = 16), affected condyle of unilateral JOA (Aff-Uni) (n = 36), non-affected condyle of JOA (NonAff-uni) (n = 36), and bilateral JOA (Bilateral) (n = 28). The differences in condyle volume and length and AE were analyzed using ANOVA and Bonferroni post-hoc tests. The results showed that Bilateral had a significantly different condylar volume, especially in the condylar head (*p* < 0.01), specifically the middle, anterior, and medial parts (*p* < 0.05). Condylar length also differed among the groups, with differences observed between the control group and the other three groups, as well as between the bilateral group and the other three groups (*p* < 0.01). AE total volume differed between the control group and Aff-Uni. In the detailed comparison, Aff-Uni and NonAff-Uni were smaller than the control group in the posterior, lateral, and medial sections (*p* < 0.05). In conclusion, depending on the involvement of unilateral or bilateral JOA, there were differences in condylar volume and AE when compared to the normal control group. Therefore, a prognosis should be evaluated by distinguishing between patients with unilateral and bilateral JOA.

## 1. Introduction

Temporomandibular disorder (TMD) is a disease that occurs in the temporomandibular joint (TMJ), surrounding structures, and masticatory muscles. Although the prevalence of TMD varies from study to study, it shows a U-shaped pattern with increasing age. Among them, TMJ osteoarthritis (OA) is a degenerative joint disease that occurs in the TMJ and is common even at a young age. The TMJ is an important growth point for facial profile formation, and growth is completed at the age of 16 for females and 18 for males on average [1]. Therefore, severe OA in juvenile patients changes the normal growth pattern and, when occurring unilaterally, results in facial asymmetry. Bilateral occurrence can cause facial deformities such as posterior rotation and mandibular retraction. In addition, the limitation of physical functions, such as mastication and conversation, makes daily functions difficult [1,2]. Therefore, early diagnosis is important, but in the early stages, clinical symptoms, such as pain and crepitation, often do not coincide with the state of arthritis, so it is often missed [3]. Therefore, the condition is accidentally discovered after visiting a hospital for orthodontic treatment because of severe facial asymmetry or for an open bite or other dental treatment such as tooth extraction and restorative treatment. However, early arthritis may not be detected on conventional images, such as panoramic images, even after visiting a hospital; therefore, it may be missed unless conebeam CT (CBCT) is performed.

In this study, juvenile idiopathic osteoarthritis (JOA) encompassed all OA in juvenile patients. In general, juvenile idiopathic arthritis (JIA) can be distinguished from idiopathic condylar resorption (ICR) as it lacks joint inflammation and synovitis. However, it is difficult to clinically differentiate between JIA and ICR that involves the TMJ with minimal pain, a lack of swelling, and low-sensitivity MRI. Both conditions show high overlap in radiological and clinical manifestations and are used interchangeably in studies [4,5,6]. Some argue that the diagnostic difference between ICR and JIA is due to the difference in the timing of diagnosis rather than the difference in the disease itself [7]. Although many studies have been conducted, an overall understanding of JOA is still limited. In addition, previous studies on condylar volume have mainly been conducted on Caucasians, and studies on Asians are lacking [8,9]. Most studies have focused on the condyle, and there are few studies on articular eminence. Therefore, in this study, we aimed to compare the volume and length of the normal condyle, unilateral and bilateral JOA, and articular eminence.

## 2. Materials and Methods

In this retrospective case-control study, eminence, condylar volume, and length were analyzed by comparing those patients affected by unilateral and bilateral JOA and comparing them with healthy controls. Written informed consent was obtained from all patients or their parents, depending on their age at the initial visit. This study was approved by the Institutional Review Board of Pusan National University Dental Hospital (IRB No. PNUDH-2021-022). This study was complied using the principles of the Declaration of Helsinki for Human Studies. 

### 2.1. Study Participants and Design

All subjects were recruited from among those who visited the Department of Oral Medicine at the Pusan National University Dental Hospital between January 2017 and December 2020. Patients who met the criteria for an OA diagnosis according to The Diagnostic Criteria for Temporomandibular Disorders (DC/TMD) were included [10,11]. As a control group, CBCT was performed for tooth extraction or orthodontic evaluation at the first visit. Patients who had no erosion, sclerosis, subcortical cysts, or flattening in both the TMJ and eminence on CBCT, no previous orthodontic treatment, and no previous craniofacial trauma were included. Patients with no congenital craniofacial deformities, condylar fractures, or TMJ tumors were also included. A total of 116 participants, including 16 control participants, were divided into four groups. (1) Control group (n = 16); (2) affected condyle of unilateral JOA (Aff-Uni) (n = 36); (3) unaffected condyle of JOA (NonAff-Uni) (n = 36); (4) Bilateral JOA (Bilateral) (n = 28). Only the right side of the subject was investigated for all joints.

### 2.2. Image Acquisition and 3D Reconstruction

CBCT images were obtained from all patients using Pax-Zenith 3D (Vatech, Hwaseong, Republic of Korea). The CBCT data were reformatted using three-dimensional (3D) imaging soft-ware (Ondemand 3-D; Cybermed Co., Seoul, Republic of Korea) set to the following parameters: field of view, 20 × 20 cm; tube voltage:110 kVp; tube current: 4.0 mA; scan time, 24 s. All interpretations were performed under the same graphical conditions. All condyles and articular eminences were visualized in the recommended bone density range (range of grayscale, from −1350 to 1650), as previously described, and were then graphically isolated prior to the 3D and volumetric measurements. The adaptive threshold was determined to be 500 Hounsfield units [9].

### 2.3. Measurements

#### 2.3.1. Condylar

**Figure 1:**
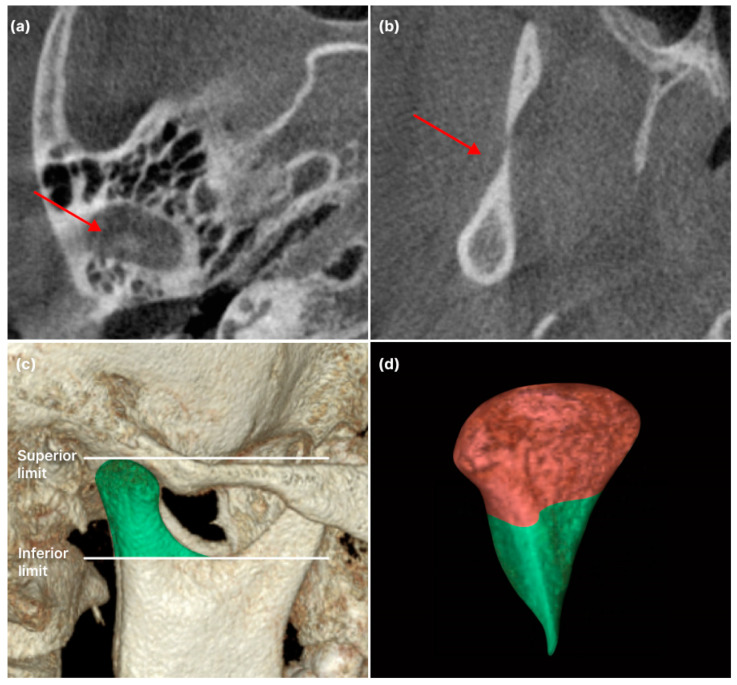
Defining boundaries for condyle volume measurement: (**a**) The superior limit of the condyle is identified when the first radiopaque point is observed in the upper articular region; (**b**) the inferior limit of the condyle is selected when the first sigmoid notch disappears; (**c**) Sagittal view, superior and inferior limit; (**d**) the head and neck of the condyle are separated at the boundary where the contour of the condyle changes from “ellipsoidal” to “circular”.

**Figure 2:**
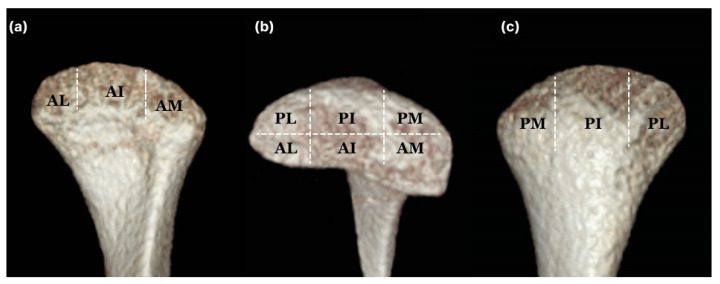
Detail volume of a condyle. Each is isometrically partitioned into the lateral, intermediate, and medial segments, thereby delineating the anterolateral (AL), anterior-intermediate (AI), anteromedial (AM), posterolateral (PL), posterior-intermediate (PI), and posteromedial (PM) sections in the head of the condyle: (**a**) anterior view; (**b**) superior view; (**c**) posterior view.

**Figure 3:**
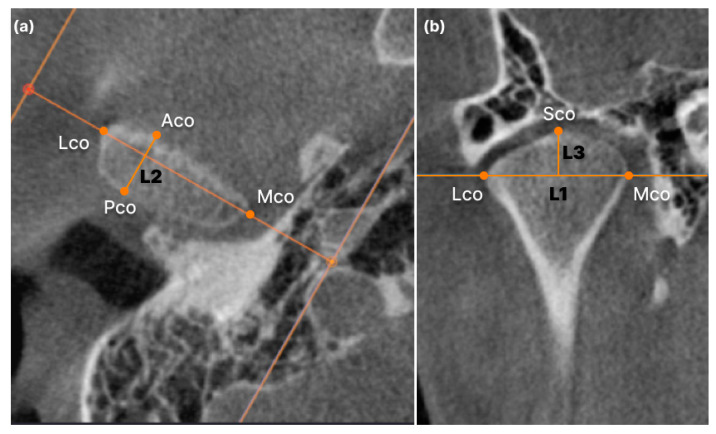
Linear measurement of condyle. Aco: most anterior extent of the mandibular condyle was defined; Pco: most posterior extent of the mandibular condyle; Lco: most lateral extent of the mandibular condyle; Mco: most medial extent of the mandibular condyle; Sco: most superior aspect of the mandibular condyle: (**a**) L2 (Aco-Pco): condylar length measured in the axial view. Linear distance between the anterior and posterior; (**b**) L1 (Lco-Mco): condylar width measured in the coronal view. Linear distance between the lateral and medial L3 (SCo-L1 perpendicular): Condylar height measured in the coronal view. Linear distance of the perpendicular line traced from Sco to L1.

The method for measuring condylar volume referred to previous studies [8,12]. Segmentation and condylar lateral limit identification were completed in the axial view. The upper limit of the condyle was considered when the first radiopacity was observed in the upper articular region. The lower limit of the condyle was selected when the first sigmoid notch disappeared. For detailed condylar volume measurements, the head and neck of the condyle were separated at the boundary where the contour of the condyle changes from “ellipsoidal” to “circular” (Figure 1). Subsequently, the plane passing through the medial and lateral poles was divided into anterior and posterior planes. Each was isometrically partitioned into lateral, intermediate, and medial segments, thereby delineating the anterolateral (AL), anterior-intermediate (AI), anteromedial (AM), posterolateral (PL), posterior-intermediate (PI), and posteromedial (PM) sections in the head of the condyle (Figure 2). Linear condyle measurements were performed using the coronal and axial views of the CBCT images. The most anterior extent of the mandibular condyle was defined as ACo, the most posterior extent of the mandibular condyle as PCo, the most lateral extent of the mandibular condyle as LCo, the most medial extent of the mandibular condyle as Mco, and the most superior aspect of the mandibular condyle as SCo. L1 (LCo-MCo): condylar width measured in the coronal view. Linear distance between the lateral and medial L2 (ACo-PCo): Condylar length measured on the axial view. Linear distance between the anterior and posterior L3 (SCo-L1 perpendicular): Condylar height measured in the coronal view. Linear distance of the perpendicular line traced from SCo to L1 (Figure 3) [13].

#### 2.3.2. Anteroposterior Condylar Position in Glenoid Fossa

**Figure 4:**
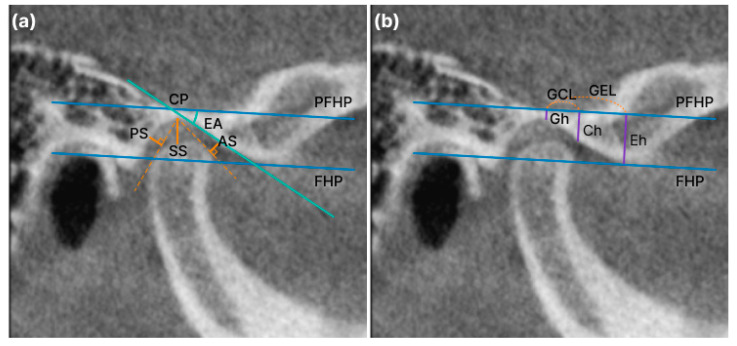
Anteroposterior condylar position in the glenoid fossa and linear measurement of the articular eminence (AE): (**a**) the anterior, posterior, and superior spaces (AS, PS, and SS, respectively) are measured from the most prominent points on the condyle to the glenoid fossa. FHP: Frank-horizontal (FH) plane; PFHP: parallel to the FHP; EA (angle): the angle at which the tangent of the distal slope passing the CP and FHP meets, that is, the articular inclination; (**b**) Gh, the length of a line perpendicular to the uppermost point of the glenoid fossa in the FHP; Eh, a line constructed parallel to the FHP tangent to the apex of the AE with a line perpendicular to the depth of the mandibular fossa; CP, the inflection point of the slope of the AE; Ch, the length of a line perpendicular to the CP in the PFHP; GEL, the distance between the PFHP and Gh points and the PFHP and Eh points; GCL, the distance between the FHP and Gh points and the FHP and CP points.

To confirm the positional relationship between the condylar positions in the glenoid fossa, the anterior, posterior, and superior spaces (AS, PS, and SS, respectively) were measured from the most prominent anterior, posterior, and superior points of the condyle to the glenoid fossa. This plane (parallel to the FH plane) was used as the reference. The lines tangential to the most prominent anterior and posterior aspects of the condyle were drawn from the superior aspect of the glenoid fossa on the reference plane (Figure 4a) [14].

#### 2.3.3. Articular Eminence

**Figure 5:**
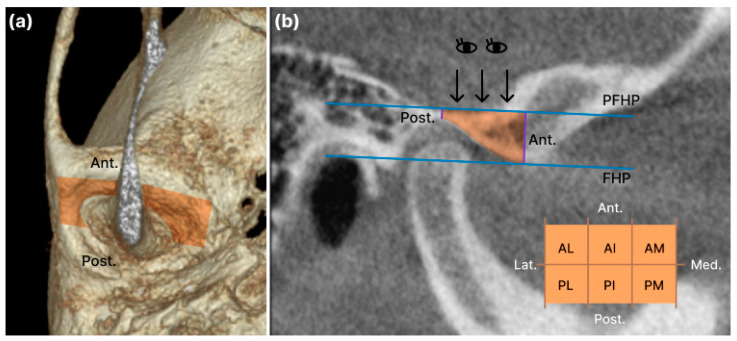
The volume of articular eminence (AE): (**a**) the boundaries of the AE are outlined in orange and are shown in the coronal view; (**b**) detailed volume measurement of the AE, anterolateral (AL), anterointermediate (AI), anteromedial (AM), posterolateral (PL), posterior-intermediate (PI), and posteromedial (PM). The eminence is divided into six equal parts.

The articular eminence (AE) volume was set as the anterior and posterior limits of the plane containing the line drawn vertically from the highest point of the glenoid fossa on the Frankfort horizontal plane and the parallel plane containing the line drawn from the lowest point of the AE on the FH plane. The lateral boundary was defined as the sagittal plane perpendicular to the FH plane passing through the outermost point of the temporal bone, whereas the medial boundary was defined as the sagittal plane perpendicular to the FH plane passing through the innermost point, where the lower boundary intersects the temporal bone. The two lines of the anterior and posterior borders and the lateral and medial borders formed a rectangle perpendicular to them (Figure 5) [15,16]. For a detailed volume measurement of the AE, the center points of each of the lateral and medial planes were connected, and the plane perpendicular to the FH plane was divided into anterior and posterior planes. Additionally, the area perpendicular to the plane dividing the anterior and posterior was divided equally into the anterolateral (AL), anterior-intermediate (AI), anteromedial (AM), posterolateral (PL), posterior-intermediate (PI), and posteromedial (PM). The eminence was divided into six equal parts (Figure 5).

#### 2.3.4. Linear Measurement of the AE

Gh was defined as the length of a line perpendicular to the uppermost point of the glenoid fossa in the FHP, and Eh was defined as a line constructed parallel to the FHP, tangent to the apex of the AE, with a line perpendicular to the depth of the mandibular fossa. The CP represents the inflection point of the slope of the AE. Ch is defined as the length of the line perpendicular to CP in the PFHP. The GEL was defined as the distance between the PFHP and Gh points and the PFHP and Eh points, and the GCL was defined as the distance between the FHP and Gh points and the FHP and CP points (Figure 4b). The EA (angle) is the angle at which the tangent of the distal slope passing through the CP and FHP meets, that is, the articular inclination. (Figure 4a)

The CBCT scans were performed by the same surgeon. The condyle and AE length and volume measurements were performed by two people and were measured again 2 weeks after the initial measurement to measure the error between the intra-observer and inter-observer methods.

### 2.4. Statistical Analysis

The data were analyzed using SPSS software (version 23.0; SPSS Inc., Chicago, IL, USA). Statistical significance was set at *p* < 0.05. 3. The Shapiro–Wilk test was used to assess whether the data were normally distributed. The chi-square test was used to analyze the sex distribution. The analysis of variance (ANOVA) and Bonferroni post-hoc were used to identify the differences in the clinical characteristics of age, NRS, MCO, overjet, and overbite. In addition, the ANOVA and Bonferroni post-hoc tests were used to evaluate the differences in volume, length, and position between the control, unilateral JOA, and bilateral JOA groups. Intraclass correlation coefficients (ICCs) for a comparison between the groups were calculated to compare the intra- and inter-operator variability.

## 3. Results

Among all the patients with JOA (n = 100), there were 76 females and 24 males: approximately three times as many females. The NRS was 3.51 ± 2.28 on average for all JOAs, which was smaller than the control group at 4.41 ± 2.34, but there was no statistical difference. In addition, there were no statistically significant differences in the clinical manifestations such as sex, NRS, age, MCO, overjet, and overbite among the four groups (Table 1).

### 3.1. Comparison of Condylar Volume and Length

Table 2 compares the condylar volume and length between the Control, Aff-Uni, NonAff-Uni, and Bilateral groups. The total condyle volume showed a statistically significant difference between the four groups, and the post-hoc test showed that it was larger for the control than for the bilateral group. After removing the neck from the condylar total volume (CTV), the condylar head total volume (CHTV) was greater in the Control and NonAff-Uni groups than in the bilateral controls. In the detailed volume measurement, a difference in volume between the groups was observed in the ants. and the post., mid, and ant. med. groups. The condylar lengths were different in L1, L2, and L3. In L1, there was a statistical difference between the bilateral and control groups, and in L2, the control group was longer than the Aff-Uni, NonAff-Uni, and Bilateral groups. At L3, the Control, Aff-Uni, and NonAff-Uni groups showed bilateral differences (Table 2).

### 3.2. Anteroposterior Condylar Position in Glenoid Fossa

There was no significant difference between the four groups in the condylar position within the glenoid fossa, but the AS was narrower in the NonAff-Uni group than in the control group (Table 3).

### 3.3. Comparison of Eminence Volume and Length for the Controls and JOA with Unilateral and Bilateral Eminence

Table 4 compares the eminence volume and length between the four groups. There was a statistically significant difference in ETV between the four groups, and the control was greater than the Aff-Uni. When comparing the size of the detailed volume, the size of the PL and PM were larger than that of the Aff-Uni and NonAff-Uni in the control group. The average eminence slope was 48.72 ± 11.53, with no statistical difference between the four groups. The lengths of Gh, Ch, Eh, GCL, and GEL were not statistically different among the four groups.

### 3.4. Results Variability

High concordance was observed in the volume and length measurements between the intra-and inter-operators. In intra-operator ICC, observer 1 was average 0.964 ± 0.020 (range: 0.923–0.986) and observer 2 was average 0.952 ± 0.017 (0.931–0.978). The inter-operator ICC was 0.912–0.974, with an average value of 0.950 ± 0.021.

## 4. Discussion

Previous studies have mainly examined condylar total volume; few studies have divided and confirmed AE and condylar volumes, as in this study [9,17,18,19,20]. The TMJ articular surface with fibrocartilage has a remarkable adaptive capacity. However, functional demands that exceed the adaptive capacity cause a maladaptive response and OA. The etiology of TMJ-OA is complex, multifactorial, and still unknown; previous studies have shown that excessive mechanical load on normal articular cartilage, or normal mechanical load on damaged articular cartilage, generally leads to OA starting from the disruption of cartilage matrix homeostasis [21]. Whenever functions such as opening and mastication are performed, the condyle and AE receive mutual pressure and rub against each other. The degree or aspect of destruction shows individual differences. Growing condyles show different adaptability to overload when compared to that observed in the adult TMJ, and this inhibits growth itself. Excessive pressure during growth can slow endochondral growth velocity, preventing cortical lining formation. This affects immature subarticular and secondary cartilages, which alters the growth direction in patients with JOA [17,21]. As a result, in this study, all the lengths of L1 (lateral and medical), L2 (anterior and posterior), and L3 (condylar height) were small, and the total condylar volume was small (Table 1).

Interestingly, in this study, there was a clear difference between the control and bilateral JOA in the condylar volume. The difference was more visible in the most convex part, which is the middle of the ant., post, and ant. sections. In the AE, a difference in size was shown in the posterolateral and medial parts of the Control, Aff-Uni JOA, and NonAff-Uni JOA groups. In the studies comparing morphological changes in condylar OA in adults, the superior surface showed the greatest difference when compared to healthy controls, as in this study [22,23].

The lateral surface of condyles usually demonstrates resorption in TMJ-OA patients, resulting in flattening on the PL section of the condylar [24,25]. From the viewpoint of homeostasis and adaptability, in patients with bilateral JOA, the condyle was relatively more destroyed along the main movement path of the joint amid mutual friction between the condyle and the AE. In the cases of Aff-Uni JOA and NonAff-Uni JOA, the lateral and medial part of posterior AE was confirmed to be more destroyed. This is likely the result of a muscle imbalance causing unilateral JOA; as a result, the direction of pressure applied to the joint is concentrated on both the lateral and medial sides rather than the middle [26,27]. Even in the positional relationship of the condyles within the glenoid fossa, this difference in unilateral JOA caused a midline shift to the affected side, resulting in a small AS in the NonAff-Uni group (Table 3).

A problem in treating patients with JOA is the mismatch between clinical symptoms and the severity of bone destruction. Therefore, many adolescent patients do not realize the seriousness of their condition, do not modify their behavior, forget to take their medicine, and do not wear the device. Therefore, the seriousness must be emphasized every time they are followed up. In that case, it is thought that the co-operation of treatment can be increased by emphasizing the reduction in bone volume, which can occur when JOA is neglected. 

Although this study provided information on condylar and AE volumes and lengths of unilateral and bilateral JOA in Asian patients, it has several limitations. First, cross-sectional conditions were compared, and the bone changes in the patients with JOA were not measured longitudinally within the same patient. In addition, in the same patient with unilateral JOA, the sample size per group decreased because the groups were divided differently by unifying all the measured joints to the right side without measuring the affected and non-affected sides within the same patient that could be affected by each other.

## 5. Conclusions

The study discovered a significant difference in condyle volume between the control and bilateral JOA groups. This difference was mainly present in the mid and medial regions of the condyle. The total length of the condyle showed L1 to be shorter in the bilateral group than in the control grouping and showed L2 to be shorter in all three groups when compared to the control group. The bilateral group demonstrated a noticeably lesser L3 value than the other groupings. Regarding the overall AE volume, patients with Aff-Uni exhibited notably lower volumes than the control group. A detailed examination of the PL and PM sections revealed similar results for both the affected and unaffected sides in terms of AE volume. In contrast to the condyle, there were no statistically significant differences observed in the AE volumes between the control and bilateral cases. Unlike previous studies that have mainly focused on the condyle volume in JOA patients, this study examined the differences in the volume of AE. The study shows that the unilateral and bilateral cases of JOAs differ in terms of the resorption of condyle and AE volumes. These findings could prove useful in clinically explaining the course of the disease to patients. Future studies ought to examine AE and condyle volume separately for unilateral and bilateral JOA.

## Figures and Tables

**Table 1:** Clinical manifestation.

		Control (n = 16)	Aff-Uni (n = 36)	NonAff-Uni (n = 36)	Bilateral (n = 28)	*p*-Value
Sex	F	8 (50.0)	28 (77.8)	24 (66.7)	24 (85.7)	0.055
	M	8 (50.0)	8 (22.2)	12 (33.3)	4 (14.3)	
Age		15.44 ± 1.75	14.06 ± 2.60	14.36 ± 2.11	14.29 ± 2.32	0.246
NRS		4.41 ± 2.34	3.44 ± 2.44	3.75 ± 2.14	3.27 ± 2.28	0.413
MCO		33.38 ± 11.41	36.97 ± 11.75	35.31 ± 7.63	33.96 ± 7.80	0.526
overjet		4.99 ± 1.59	4.92 ± 2.27	4.41 ± 2.67	5.46 ± 2.06	0.351
overbite		0.38 ± 3.56	1.39 ± 2.44	2.14 ± 2.09	1.14 ± 2.79	0.153

JOA, juvenile idiopathic osteoarthritis; Aff-Uni, affected condyle of unilateral JOA; NonAff-Uni, non-affected condyle of JOA; Bilateral, bilateral JOA; NRS: numeric rating scale; MCO: maximum comfort opening; *p*-value was obtained by chi-square test and ANOVA test.

**Table 2:** A comparison of condylar length and volume for the controls and unilateral and bilateral JOA.

	Control (n = 16)	Aff-Uni (n = 36)	NonAff-Uni (n = 36)	Bilateral (n = 28)	*p*-Value	Post-hoc (Bonferroni)
CTV	1772.51 ± 631.34	1511.20 ± 608.02	1645.34 ± 521.54	1290.00 ± 552.59	0.030 *	Controls > Bilateral
CHTV (mm^3^)	1245.23 ± 408.75	1003.70 ± 370.08	1120.17 ± 374.95	843.57 ± 326.02	0.003 **	Controls, NonAff-Uni > Bilateral
CAntLatV	276.28 ± 139.81	262.43 ± 137.75	264.49 ± 121.81	230.25 ± 131.03	0.643	
CPostLatV	87.99 ± 54.69	64.45 ± 31.25	80.24 ± 39.84	56.53 ± 41.39	0.029	
CAntMidV	521.83 ± 184.08	397.58 ± 201.12	436.29 ± 199.03	320.29 ± 150.67	0.006 **	Controls > Bilateral
CPostMidV	166.80 ± 98.98	137.24 ± 70.93	172.96 ± 69.73	115.94 ± 55.48	0.010 **	NonAff-Uni > Bilateral
CAntMedV	139.23 ± 80.55	91.17 ± 46.59	105.87 ± 74.83	76.91 ± 45.56	0.012 *	Controls > Bilateral
CPostMed	53.11 ± 21.94	50.83 ± 29.05	60.32 ± 37.40	43.66 ± 25.16	0.186	
L1 (mm)	19.35 ± 2.49	17.49 ± 2.69	17.38 ± 2.80	16.20 ± 2.86	0.005 **	Controls > Bilateral
L2 (mm)	19.16 ± 2.46	17.08 ± 2.68	16.80 ± 2.44	15.74 ± 2.08	0.000 ***	Controls > Aff-Uni, NonAff-Uni, Bilateral
L3 (mm)	5.75 ± 1.51	5.28 ± 1.39	5.96 ± 1.29	4.35 ± 1.05	0.000 ***	Controls, Aff-Uni, NonAff-Uni > Bilateral

JOA, juvenile idiopathic osteoarthritis; Aff-Uni, affected condyle of unilateral JOA; NonAff-Uni, non-affected condyle of JOA; Bilateral, bilateral JOA; CTV, Condyle total volume; CHTV, Condyle head total volume; CAntLatV, condyle anterior lateral volume; CPostLatV, condyle posterior lateral volume; CAntMidV, condyle anterior middle volume; CPostMidV, condyle posterior middle volume; CAntMedV, condyle anterior medial volume; CPostMed, condyle posterior medial volume; L1, condylar width measured on the coronal view. Linear distance between lateral and medial, L2, and condylar length measured on the axial view. Linear distance between the anterior and posterior, L3, condylar height measured on the coronal view; *p*-value was obtained by ANOVA test and Bonferroni post-hoc; * *p* < 0.05; ** *p* < 0.01; *** *p* < 0.001.

**Table 3:** Anteroposterior condylar position in glenoid fossa.

	Control (n = 16)	Aff-Uni (n = 36)	NonAff-Uni (n = 36)	Bilateral (n = 28)	*p*-Value	Post-hoc (Bonferroni)
PS	1.97 ± 0.68	2.04 ± 1.34	2.06 ± 1.15	2.19 ± 1.24	0.943	
SS	3.38 ± 1.12	2.79 ± 1.22	2.58 ± 0.80	2.98 ± 1.53	0.146	
AS	3.94 ± 1.80	3.29 ± 1.81	2.63 ± 1.08	3.21 ± 1.46	0.036 *	Controls > NonAff-Uni

JOA, juvenile idiopathic osteoarthritis; Aff-Uni, affected condyle of unilateral JOA; NonAff-Uni, non-affected condyle of JOA; Bilateral, bilateral JOA; PS, posterior space; SS, superior space; AS, anterior space. *p*-values were obtained by ANOVA and Bonferroni post-hoc tests; * *p* < 0.05.

**Table 4:** A comparison of eminence length and volume for the controls and unilateral and bilateral JOA.

	Control (n = 16)	Aff-Uni (n = 36)	NonAff-Uni (n = 36)	Bilateral (n = 28)	*p*-Value	Post-hoc (Bonferroni)
EA (angle)	54.36 ± 10.35	49.49 ± 11.89	46.86 ± 11.60	46.84 ± 11.02	0.130	
Gh (mm)	2.16 ± 1.47	1.60 ± 1.11	1.96 ± 1.91	2.01 ± 1.22	0.558	
Ch	6.39 ± 1.68	6.00 ± 1.44	6.16 ± 2.12	6.50 ± 1.45	0.684	
Eh	10.33 ± 1.95	9.32 ± 1.82	9.80 ± 2.39	9.58 ± 1.57	0.376	
GCL	5.11 ± 1.61	5.39 ± 1.23	5.44 ± 1.34	6.18 ± 1.91	0.084	
GEL	11.14 ± 3.25	11.38 ± 1.73	12.29 ± 2.06	12.57 ± 2.38	0.072	
ETV	254.97 ± 174.42	150.89 ± 76.30	168.70 ± 120.55	202.24 ± 119.33	0.023 *	Controls > Aff-Uni
EAntLatV	28.90 ± 23.34	14.28 ± 12.14	22.08 ± 28.77	22.19 ± 27.88	0.197	
EAntMidV	43.64 ± 33.97	29.36 ± 19.86	32.68 ± 36.02	38.42 ± 32.80	0.394	
EAntMedV	41.72 ± 29.80	24.94 ± 17.06	29.21 ± 22.55	31.21 ± 25.90	0.118	
EPostLatV	46.54 ± 37.75	23.69 ± 14.32	27.91 ± 22.07	33.20 ± 20.07	0.008 **	Controls > Aff-Uni, NonAff-Uni
EPostMidV	49.53 ± 36.71	33.16 ± 18.86	31.30 ± 19.71	42.68 ± 26.11	0.036	
EPostMedV	44.64 ± 34.09	25.46 ± 13.64	25.52 ± 18.06	34.54 ± 25.23	0.011 *	Controls > Aff-Uni, NonAff-Uni

JOA, juvenile idiopathic osteoarthritis; Aff-Uni, affected condyle of unilateral JOA; NonAff-Uni, non-affected condyle of JOA; Bilateral, bilateral JOA; FHP, frankfort horizontal plane; PFHP, parallel line to Frankfort horizontal plane; CP, inflection point of the articular eminence (AE) slope; EA(angle), Angle at which the tangent of the distal slope passing through CP and FHP meet; Gh, Length of the perpendicular line from the most superior part of the glenoid fossa to PFHP; Eh, PHFP to the apex of the AE with a perpendicular constructed from this line to the depth of the mandibular fossa; Ch, Length of the perpendicular line from PHFP to CP; GEL, Distance between PFHP and Gh point and PFHP and Eh point; GCL, Distance between FHP and Gh points and FHP and CP points; ETV, AE total volume; EAntLatV, anterolateral volume of eminence; EAntMidV, anteromiddle volume of eminence; EAntMedV, anteromedial volume of eminence; EPostLatV, posterolateral volume of eminence; EPostMidV, posteromiddle volume of eminence; EPostMedV, posteromedial volume of eminence; *p*-value was obtained by ANOVA test and Bonferroni post-hoc; * *p* < 0.05; ** *p* < 0.01.

## Data Availability

The data used to support the findings of this study are included within the article.
